# Clinical performance of an automated reader in interpreting malaria rapid diagnostic tests in Tanzania

**DOI:** 10.1186/1475-2875-12-141

**Published:** 2013-04-24

**Authors:** Seif Shekalaghe, Marcela Cancino, Caroline Mavere, Omar Juma, Ali Mohammed, Salim Abdulla, Santiago Ferro

**Affiliations:** 1 Ifakara Health Institute, Bagamoyo Research and Training Centre, PO Box 74, Bagamoyo, Tanzania; 2 Fio Corporation, Toronto, Canada

**Keywords:** Deki Reader, Malaria rapid diagnostic test, mHealth

## Abstract

**Background:**

Parasitological confirmation of malaria is now recommended in all febrile patients by the World Health Organization (WHO) to reduce inappropriate use of anti-malarial drugs. Widespread implementation of rapid diagnostic tests (RDTs) is regarded as an effective strategy to achieve this goal. However, the quality of diagnosis provided by RDTs in remote rural dispensaries and health centres is not ideal. Feasible RDT quality control programmes in these settings are challenging. Collection of information regarding diagnostic events is also very deficient in low-resource countries.

**Methods:**

A prospective cohort of consecutive patients aged more than one year from both genders, seeking routine care for febrile episodes at dispensaries located in the Bagamoyo district of Tanzania, were enrolled into the study after signing an informed consent form. Blood samples were taken for thick blood smear (TBS) microscopic examination and malaria RDT (SD Bioline Malaria Antigen Pf/Pan™ (SD RDT)). RDT results were interpreted by both visual interpretation and Deki Reader™ device. Results of visual interpretation were used for case management purposes. Microscopy was considered the “gold standard test” to assess the sensitivity and specificity of the Deki Reader interpretation and to compare it to visual interpretation.

**Results:**

In total, 1,346 febrile subjects were included in the final analysis. The SD RDT, when used in conjunction with the Deki Reader and upon visual interpretation, had sensitivities of 95.3% (95% CI, 90.6-97.7) and 94.7% (95% CI, 89.8–97.3) respectively, and specificities of 94.6% (95% CI, 93.5–96.1) and 95.6% (95% CI, 94.2–96.6), respectively to gold standard. There was a high percentage of overall agreement between the two methods of interpretation.

**Conclusion:**

The sensitivity and specificity of the Deki Reader in interpretation of SD RDTs were comparable to previous reports and showed high agreement to visual interpretation (>98%). The results of the study reflect the situation in real practice and show good performance characteristics of Deki Reader on interpreting malaria RDTs in the hands of local laboratory technicians. They also suggest that a system like this could provide great benefits to the health care system. Further studies to look at ease of use by community health workers, and cost benefit of the system are warranted.

## Background

Responding to the World Health Organization (WHO) recommendation that treatment for malaria cases should be administered only to cases with parasitological confirmation either by microscopy or rapid diagnostic tests (RDTs), many countries in sub-Saharan Africa (SSA) have adopted the use of malaria RDTs to improve case management and promote appropriate use of anti-malarial drugs [1]. The apparent simplicity of malaria RDT makes them especially attractive for diagnosis at all levels of health care systems, particularly in remote areas where health workers have limited supervision and training. The reliability of malaria RDT results is pivotal to ensuring the safety of withholding anti-malarial treatment in test-negative patients [2-5].

The possibility of error in diagnosis using RDTs can be minimized through a careful selection process of RDTs, as recommended by WHO [6]. Among others, important steps involved in the process to ensure high quality of malaria RDT include high diagnostic performance (as assessed in product testing by WHO and the Foundation for Innovative New Diagnostics [7]), proper storage conditions of the tests, adequate labelling, clear performance instructions, continuous on-the-job training (job aids), and automated interpretation to avoid inter-user variability. Previous studies have demonstrated improvement in the performance of health professionals using malaria RDTs through simple redesign of job aids [8,9].

Large-scale implementation of a diagnostic programme based on malaria RDTs requires the establishment of a comprehensive quality assurance (QA) strategy. This task can be particularly challenging considering that most RDTs are being used at point-of-care (POC) in health centres and dispensaries located in remote areas. Current recommendations by WHO include assessment of the competence of community health workers to perform RDTs, and to interpret and correctly use the results during supervisory visits, by direct observation with a standard checklist [10]. However, research has shown that although QA programmes based on direct supervision can produce good outcomes, their sustainability is highly questioned due to the costs implicated in personnel, transportation and others [11,12]. One way to overcome these additional costs is by conducting supervisory activities from a remote location, increasing the efficiency of the system as one supervisor can conduct QA activities to multiple facilities without the need of visiting the sites. Remote supervision by means of photographed HIV and malaria RDTs has been shown to give good results in preliminary studies [13-15].

Recently, the Fio Corporation (Toronto, Canada) has developed a solution based on smartphone technology consisting of a mobile, battery-operated device (Deki™Reader), with a touch screen interface, that performs automated interpretation of RDTs (universal reader) using proprietary digital image analysis software. Information related to the diagnostic event (demographics, symptoms, etc) is collected through the user interface, and after the RDT has been interpreted, the data along with a high-resolution image of the RDT are transmitted to a server using mobile-phone technology. At Fio cloud database (airFio™) the data are aggregated, analysed and presented in customized reports that can be accessed by authorized users in real time via a secure website portal.

The use of new technologies, such as the one used in the present study, can help the large-scale implementation of RDT programmes by minimizing the chance of errors in diagnosis, providing expedited reporting of all diagnostic events, including those performed at remote POC locations, and allowing close monitoring of quality performance in the field. The objective of the present study was to assess the clinical diagnostic accuracy of the SD RDT when interpreted by the Deki Reader device and to compare it to the visual interpretation of RDTs by experts for detection of *Plasmodium falciparum* infections in an outpatient population in a malaria-endemic area of Tanzania.

## Methods

### Study design and participants

This was a study conducted in a prospective cohort of consecutive patients from January to March 2012 at Bagamoyo district in Tanzania. The district is situated north of Dar es Salaam and has an estimated population of 270,000 inhabitants, all of whom are considered to be at risk to develop malaria.

Recruitment was from three peripheral health care dispensaries (Kiwangwa, Yombo, and Fukayosi) located in the rural vicinity (approximately 30 km from the town of Bagamoyo), and from Bagamoyo District Hospital outpatient clinic in the urban centre of Bagamoyo. Subjects from both genders older than one year who were seeking routine care for malaria were enrolled into the study. A patient was enrolled once they were found to meet inclusion and exclusion criteria and had signed a study specific informed consent form.

Before the conduct of the trial, the protocol was reviewed and approved by national (Ref: NIMR/R.8a/Vol.IX/l244) and institutional (Ref: IHI/IRB/No: 34) ethics committees and regulatory authority, TFDA (Ref: No: CEm57/180/04A/54).

### Malaria rapid diagnostic tests

The malaria RDT used in the current trial was SD Bioline Malaria Antigen Pf/Pan (Catalogue No. 05FK60, Standard Diagnostics Inc, Hagal-Dong, Korea, from now on referred as “SD RDT”). This is a lateral flow immunochromatographic test that contains a membrane strip encased in a flat plastic cassette. The strip is precoated with two antibodies: one that is specific for *P*. *falciparum* HRP2 and one that is pan-specific for pLDH for detection of other *Plasmodium* species.

### The Deki Reader™

**Figure 1:**
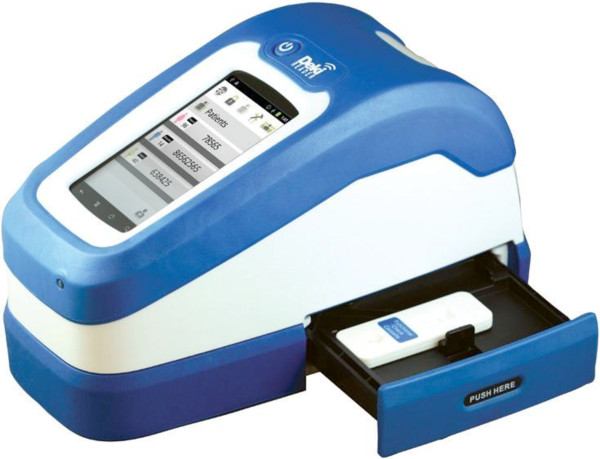
**Deki Reader**^**TM **^**device.**

This mobile, rugged, battery-operated device (Fio Corporation, Toronto, Canada) performs the following functions (see Figure 1):

1. Automated interpretation of RDTs by means of image analysis software;

2. Digital data capture, by means of a touch-screen and a simple user interface software;

3. Transmission in real time using local mobile phone network of processed RDT image, diagnostic event data collected, geopositioning of the device, and date and time stamp to a central database, which is accessible via internet.

### Procedures

Eligible participants were consecutively enrolled in the dispensaries and at the outpatient clinic in Bagamoyo District Hospital. Inclusion criteria were age over one year, a documented fever or a history of fever in the previous 48 hours and consent to participate in the study. No information regarding recent self-treatment with anti-malarials was collected. Clinicians enrolled study participants and collected demographic and clinical information using a structured form.

After enrolment and physical examination by the study clinician, blood was collected from a finger prick once (no follow ups) and about three to four drops were collected in a heparinized microtube and sent to the reference laboratory of Ifakara Health Institute in Bagamoyo for preparation of thick blood smear (TBS) and a filter paper blood spot for polymerase chain reaction (PCR) testing, to be performed as quality control of microscopy. Technicians working at the reference laboratory were blinded to results of both visual and device interpretation of malaria RDTs performed at the site. Results of TBS microscopy examination and PCR (when applicable) were blinded to site technician who performed RDTs. At each of the study sites, the study technician collected blood from a finger prick for the processing of SD RDTs according to manufacturer’s recommendations. The processing of the RDT was guided by a job aid displayed on the screen of the Deki Reader device. The device also assisted technicians at the sites in keeping track of the incubation time for each RDT processed. Once the incubation period was completed, the study technician interpreted the RDT by visual inspection of the strip and captured the result using the touchscreen of the device. The study technician then immediately inserted the cassette into the device to perform the automated interpretation of the test. The results of the device’s interpretation of the RDT were concealed at all times, and were generated in all cases after the study site technician had entered results of her/his visual interpretation of RDT test results. All results of interpretation of RDTs, along with a high-resolution image of the RDT, and patient data collected were encrypted and automatically transmitted to a central cloud database specifically designed for the purpose of the trial.

Patient management was performed according to routine local protocols, involving results of RDTs after visual interpretation by the study technician, and the opinion of the clinician at the site.

### Laboratory analyses

TBS microscopy and PCR tests were performed at Ifakara Health Institute Reference Laboratory, Bagamoyo by technicians who were blinded to results of RDT interpretation by both expert study technicians and the Deki Reader.

At the reference laboratory, blood smears were stained using 10% Giemsa solution for 30 minutes and examined under high-power objective. In positive smears, asexual and sexual parasites were counted against 200 and 500 white blood cells (WBCs), respectively. Parasite density was obtained by multiplying the parasite counts by 40 for asexual and 16 for sexual parasites (assuming each μL of blood contained 8,000 WBCs). A smear was declared negative after examining 200 high-power fields.

PCR was performed in all cases of discrepant results between expert visual interpretation of RDT, device’s interpretation of RDT and expert microscopy results (tiebreaker); and in a randomly selected subset of negative and positive samples (quality control of TBS microscopy). The PCR technique has high analytical sensitivity, allowing for qualitative and quantitative demonstration of parasites in clinical samples.

Molecular analysis involved extraction of parasite DNA from dried blood spots on filter papers. The DNA was used to determine the presence of *P*. *falciparum* beta tubulin target gene. DNA was extracted from dried blood spots by using QIAamp DNA mini kit (Qiagen), and PCR amplification was performed using Invitrogen QPCR SuperMix-UDG kit (Invitrogen). Real-time PCR amplification was done using MxPro3005 QPCR System (Agilent Technologies Inc, USA); forward (5’-TGA TGT GCG CAA GTG ATC C-3’) and reverse (5’-TCC TTT GTG GAC ATT CTT CCT C-3’) primers hybridize to the pathogen DNA. A probe (5’-TA GCA CAT GCC GTT AAA TAT CTT CCA TGT CT-3’) is included in the same reaction mixture. During PCR amplification, if the DNA of interest was present and amplified, the probe was cleaved and the reporter dye and quencher were separated leading to production of fluorescence. At the end of each PCR cycle, the resulting increase in fluorescence was detected by qPCR system as fluorescence units. As amplification continued, the cycle number at which detection of DNA products (threshold cycle value) was reached, and this information together with the standards (3D7 200,000 p/μl; 20,000 p/μl; 2,000 p/μl; 200 p/μl; 20 p/μl) gave the value of parasitaemia.

### Statistical analysis

Data were obtained from source documents at each of the participant sites and compared to information collected through devices and transmitted to the Fio cloud database. Data collected were monitored according to FDA regulations and GCP guidelines. There were three outcomes of interest for the purpose of the analysis in the current trial: a) visual interpretation of RDT: performed by laboratory technician at the site following visual inspection of the SD RDT at the appropriate time. This information was entered by the technician into the device; b) device interpretation of RDT: performed by the device, which was collected automatically by the device and transmitted to the database; and, c) microscopy results: performed on TBS by laboratory technician at reference laboratory and entered into the dataset manually. For the purpose of the analysis the result of TBS microscopy was considered the “gold standard”.

Data collected at the Fio database were compared to data collected in the internal memory card of each Deki Reader device to ensure all data points were correctly transmitted to the cloud database. Data analysis was performed using JMP Version 8.0.2 and R Version 2.12.1 (SAS Institute Inc, NC, USA).

The primary analysis was conducted using TBS microscopy as gold standard to determine diagnostic performance characteristics (sensitivity, specificity, negative (NPV) and positive (PPV) predictive values, and overall diagnostic accuracy) of RDT interpretation by the Deki Reader and by visual interpretation. Correspondent 95% confidence intervals were constructed using the Wilson score method.

The percentage of agreement in the interpretation of SD RDT between the device and visual (human eye) done by the expert laboratory technicians at the sites was calculated as percentage of negative agreement, percentage of positive agreement and overall percentage of agreement. Two-sided 95% confidence intervals were constructed using the Wilson score method.

### Sample size estimate

Although the RDT used in the present study is able to detect malaria infections caused by all *Plasmodium* species (categorized as “P.f” and “Pan”), the statistical analysis was performed only in results of *P*. *falciparum* test line, due to the small number of infections expected by parasites other than *P*. *falciparum*.

The prevalence of malaria among febrile patients presenting to a health clinic in the study area was estimated to be 10 to 15%, with greater than 95% of malaria cases caused by *P*. *falciparum*.

Based on this prevalence of *P*. *falciparum* infection in the study area, the sample size was estimated to be between 1,200 to 1,500 subjects, under the assumption that it would provide approximately 120-225 positive results for *P*. *falciparum*. Assuming that the sensitivity and specificity of the interpretation of SD RDT is the same as the sensitivity and specificity of the human-eye interpretation this sample size would provide 95% confidence intervals within 5% of the point estimate.

## Results

**Figure 2:**
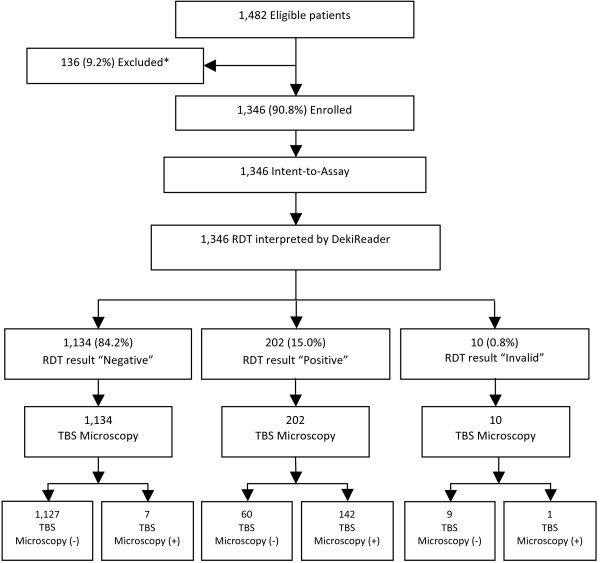
**Patient Flowchart for Deki Reader RDT result.** *Subjects were enrolled as part of a pilot run of the study, but excluded from the final analysis as planned. Intent-to-Assay: Included all enrolled subjects who met all inclusion and exclusion requirements and whose test was inserted within the 30-minute window, as per protocol.

Between January and March 2012, a total of 1,482 subjects were screened. Of the total, 1,346 (90.8%) met inclusion criteria after the pilot period and were included in the study and final analysis (Figure 2). The main demographic features of the cohort are described in Table 1. The proportion of female participants was 60.3%, and the population was predominantly children and young adolescents below the age of 17 years (64.1%) (Table 1). The prevalence of microscopically confirmed malaria was 11.1% for the full cohort, and varied among the four sites: 3.4% at Bagamoyo District Hospital, 11.9% at Yombo dispensary, 12.2% at Kiwangwa dispensary, and 15.6% at Fukayosi dispensary.

**Table 1:** Demographics of study population

**Enrolled (N=1346)**	
**Gender**	
Female	812 (60.3%)
Male	534 (39.7%)
**Age (years)**	
Mean (SD)	15.84 (16.7)
Median	10
Range	[0.04,100]
**Age categories (years)**	
<1	11 (0.8%)
≥ 1 to < 5	410 (30.5%)
≥ 5 to <17	430 (31.9%)
≥ 17 to <65	460 (34.2%)
≥ 65	35 (2.6%)

### Performance characteristics of the Deki Reader and visual interpretation of SD RDTs to gold standard

When compared to standard TBS microscopy, results in interpretation of SD RDT, the Deki Reader™, had a sensitivity of 95.3% (95% CI, 90.6-97.7) and a specificity of 94.6% (95% CI, 93.5-96.1). The sensitivity of expert visual interpretation of RDTs was 94.7% (95% CI, 89.8-97.3) and the specificity was 95.6% (95% CI, 94.2-96.6) (Table 2).

**Table 2:** **Deki Reader and Visual diagnostic performance using microscopy as **“**gold standard**”

	**N**	**Sensitivity** (**95**% **CI**)	**Specificity** (**95**% **CI**)	**PPV** (**95**% **CI**)	**NPV** (**95**% **CI**)	**Overall diagnostic accuracy** (**95**% **CI**
**Deki reader**	1346	95.3 (90.6, 97.7)	94.9 (93.5, 96.1)	70.3 (63.7, 76.2)	99.4 (98.7, 99.7)	95 (93.7, 96.0)
**Visual**	1346	94.7 (89.8, 97.3)	95.6 (94.2, 96.6)	72.8 (66.2, 78.6)	99.3 (98.6, 99.6)	95.5 (94.2, 96.5)
**Comparison**		0.7 (-2.7, 4.2)	-0.7 (-1.5, 0.1)			

### Comparison of performance characteristics of the Deki Reader to visual interpretation of SD RDTs

The percentage agreement for the interpretation of RDTs between the device and visual was 98.7% (95% CI, 93.4-98.6) when a negative result was obtained, 96.9% (95% CI, 97.8-99.2) when a positive result was obtained, and an overall percentage agreement of 98.4% (95% CI, 97.6-99.0) (Table 3). There was no statistically significant difference in the sensitivity (0.7, 95% CI, -2.7-4.2), nor in the specificity (-0.7, 95% CI, -1.5-0.1) between the two methods of interpretation.

**Table 3:** **Comparison of performance between Deki Reader and visual interpretation** (**n**= **1346**)

**Negative** % **agreement** (**95**% **CI**)	**Positive** % **agreement** (**95**% **CI**)	**Overall** % **agreement** (**95**% **CI**)
98.7 (97.8, 99.2)	96.9 (93.4, 98.6)	98.4 (97.6, 99)

### Discordant test results between Deki Reader and visual interpretation of SD RDTs

There were 29/1,346 (2.2%) discordant test results between the Deki Reader and the visual interpretation of RDTs. In eight of these cases, the software did not detect a control line, and therefore classified the test as “invalid” according to manufacturer’s recommendations. The visual interpretation of these eight cases was “negative” in six of them and “positive” in two. No re-testing of these samples was done. Out of the remaining 21 cases, in 15 the visual interpretation was reported as “negative”, and the device interpretation was “positive”; while in the other six cases, it was the other way around. In 10 (48%) of these 21 cases the discrepancy seems to have originated in a clerical error when entering the information into the device. Analysis of RDT images for these 10 cases that were transmitted to the portal and sorted in the database showed either the presence of a clearly visible control and test line(s) (and human expert interpretation was erroneously entered as “negative”) or a complete absence of test lines in the diagnostic strip (and human expert interpretation was erroneously entered as “positive”). The remaining 11 discrepant cases all correspond to RDTs visually interpreted as negative, in which the intensity of the test line was around the cut-off of the software, in some cases producing very faint lines that could have been missed by visual inspection (see Additional file 1). The PCR test results in these 11 cases showed two samples positive for *P*. *falciparum*, one of which was also positive by microscopy. Therefore, in 9/1,336 cases (0.7%) discrepant results were truly false positive results.

### Feasibility outcomes

The present field study confirmed that the Deki Reader, operated by laboratory technicians after a two-day training session, is able to collect information related to diagnostic events using malaria RDTs. The information collected, along with the correspondent RDT image, was sent immediately from remote areas to a database, being part of the Fio cloud services with 100% fidelity. Study managers were able to monitor the progress of the study at each of the participating sites by using the Fio portal to review upcoming data in real time. Access to the data was possible from different locations and permanent.

### TBS microscopy quality control

The overall agreement between TBS microscopy and PCR results was 88.9% (48/54). In three samples each of TBS positive and TBS negative results, PCR results were discordant (Table 4). Results of PCR were concordant in 87% (20/23) of TBS microscopy positive results, and in 90% (28/31) of TBS microscopy negative results.

**Table 4:** Two by two table of microscopy and PCR results

		**PCR**		
		**Positive**	**Negative**	**Total**
**Microscopy**	Positive	**20**	3	23
	Negative	3	**28**	31
**Total**		23	31	**54**

## Discussion

Strengthening of malaria control programmes and large investments in malaria prevention in SSA has resulted in significant accomplishments in the reduction of both malaria mortality and morbidity. In turn, decreasing malaria transmission has led to a decline in the proportion of fevers attributable to malaria. This fact, and the use of relatively expensive artemisinin-containing treatments as first-line anti-malarials have made it very important to provide accurate malaria diagnosis at all levels of health systems [10,16]. The WHO now recommends parasitologic confirmation in all clinically suspected cases prior to treatment [1].

Diagnosis at POC can be done by means of malaria RDTs, since they represent an accurate, affordable, relatively easy-to-use malaria diagnostic method, and in some instances have been reported to be even more reliable than microscopy to provide widespread access to diagnosis [17-19]. In order to be widely adopted, malaria RDTs must have both high (>95%) sensitivity and specificity in field settings. High sensitivity is necessary to ensure that true cases of malaria are identified and appropriately managed while high specificity is needed to avoid false positive results that would lead to missed diagnosis of the true cause of fever, but also to avoid unnecessary anti-malarial treatment. Implementation of RDT programmes at large scale is promoted by WHO [10] as part of the T3 initiative in highly endemic countries [20]. Despite their simplicity and apparent ease of processing and interpretation, there have been reports of considerable deficiencies in the processing and interpretation of RDTs in general, and malaria RDTs in particular [8,9,15], which indicates the need for a robust QA plan to be implemented along with RDT-based diagnostic programmes. A recent publication on QA strategy in rural Tanzania, based on current WHO recommendations, concluded that “Future research in RDT implementation should focus on testing QA strategies that are less labour intensive, costly, and more widely implementable. The WHO recommendation to obtain blood smears with 20 positive and 20 negative RDTs should be tested in a variety of transmission settings and other QA strategies employing positive controls, PCR, or other diagnostic methods should be explored to ensure reliable performance of RDTs in field conditions” [12].

On the other hand, over the last decade considerable effort has been dedicated to the development of automated RDT readers, utilizing diverse technologies such as digital image software analysis, and scanning software. The idea behind the use of automated readers is to minimize human error involved in processing and interpretation of RDTs, which can be considerable, as indicated above [8,9,15]. As a result, a number of devices have been introduced in the market place. However, most of them are still relatively expensive and not suitable for operation at POC conditions such as those in malaria-endemic regions of rural Africa [21].

In this study, routine microscopy was used as gold standard to assess the performance characteristics of Deki Reader, an automated RDT reader that uses digital software image analysis technology, in the interpretation of malaria SD RDTs. The performance characteristics of Deki Reader were also compared to that of visual interpretation of the SD RDTs performed by experienced laboratory technicians. The main findings are that the sensitivity and specificity of Deki Reader in the interpretation of SD RDTs were 95.3% and 94.6% respectively, while those of visual interpretation by experts were 94.7% and 95.6% respectively (Table 2). There was no statistically significant difference between the diagnostic accuracy of the Deki Reader and the visual interpretation of malaria RDTs.

Based on these results, the present study has demonstrated that RDT interpretation by Deki Reader is substantially equivalent to that of visual interpretation as performed by laboratory technicians with vast experience in RDT processing and interpretation. The fact that the results from the current study, conducted in rural health facilities and in actual clinical practice by enrolling patients who were receiving routine care for malaria, suggest that a device like the Deki Reader can be used in routine care for interpretation of RDT and therefore provide health care workers at different levels of the health care system, from community health workers to laboratory technicians to clinicians, with high-quality diagnostic results at POC. This feature will enable health care workers to make informed clinical judgments, while at the same time collecting and reporting pivotal demographic, clinical and programmatic information to health programme administrators in a central location.

The information collected during the present study was automatically encrypted, and transmitted in real time using local mobile network providers into a secured cloud database. The principal investigator and research study coordinator were able to access the information permanently using a specific password to log in to the web portal system. This way they were able to monitor field activities in each of the four participating centres, and to perform quality control activities in the processing of RDTs by conducting routine examination of the images coming from the study centres.

The results showed a relatively small percentage (2.2%) of discordant results between Deki Reader and visual interpretation in this study. A closer look at the discordant results showed that in about half the cases, the discrepancy was due to apparent clerical mistakes suggesting that device interpretation was correct; while in only nine cases (0.7%) was this due to a false positive result interpretation by the device (see Additional file 1). The importance of this finding is negligible considering that the criterion established by WHO for procurement of malaria RDTs is of less than 10% of false positive rate [6]. Nonetheless, the newest version of the software in the device has been improved and is now able to capture some of the more common causes of smearing of the diagnostic strip in the RDTs that can lead to automated interpretation of false positive results, as in some of the cases shown here. In all instances the user will be requested to repeat the test and to adhere to the manufacturer recommendations related for processing of the mRDT.

The availability of a system such as the one tested in the present study can bring significant benefits to the widespread implementation of malaria RDTs being adopted by many countries in SSA. At the POC level, the increased accuracy in diagnosis delivered by automated interpretation of RDTs could increase adherence to national treatment guidelines, decreasing the rates of overtreatment of non-malaria cases. The beneficial effects here may extend well beyond malaria, as the Deki Reader is a universal RDT reader. That means it can interpret not only multiple brands of malaria RDTs, but also RDTs for other infectious diseases such as HIV, syphilis, dengue fever, and others. The expedited flow of accurate information the system brings from peripheral dispensaries and health centres to central locations where decision-making health programme administrators are located is expected to deliver great benefits in terms of quality control of diagnostic tests and case management, assessment of overall efficiency of the health care system and inventory management.

### Limitations

One of the main limitations of the present study was to use TBS microscopy as the gold standard method. It is well known that microscopy is an imperfect subjective diagnostic method with multiple factors affecting interpretation of blood smears, such as variability in techniques of blood film preparation, staining, reading standards, and most importantly high dependency on the level of expertise of the examining microscopists [17,22-26]. Therefore, to limit these difficulties, microscopy in this study was performed by experienced technicians in a single referral centre who analysed films independently and were blinded to the RDT results.

Another limitation is in the generalization of the results of the present study since only one malaria RDT (SD Bioline Malaria Antigen Pf/Pan) was tested to assess the performance characteristics of the Deki Reader. Considering the fact that different RDTs are available and used, the results cannot be generalize to other RDTs. However, the function of the Deki Reader is to interpret the presence or absence of a test line signal, independent of the analytes that are finally deposited on it. Hence, the performance of the automated reader is independent of the underlying immunochemistry and thus should provide similar performance characteristics for other malaria RDTs using different antigens and for RDTs used in diagnosis of other diseases in general.

## Conclusions

Despite the limitations of this study, the results obtained reflect the real situation of performance characteristics of the Deki Reader on interpreting malaria RDTs. More studies are recommended to provide further evidence of current findings and on the performance of the device using other types of RDTs. Further studies looking at feasibility of the use of the Fio system at community health worker level, cost benefit, suitability and acceptability by users, as well as involving RDTs for other diseases and other brands of malaria RDTs are warranted.

## Competing interests

The authors declare that they have no competing interests. SF and MC are employees of Fio Corporation.

## Authors’ contributions

SS, SA and SF conceived and designed the study. SS, SA and SF wrote the manuscript. MC, CM, OJ and AM collected and monitored the data and provided comments on the manuscript. All authors read and approved the final manuscript.

## Supplementary Material

Additional file 1 Discordant test Results between Deki Reader and Visual Interpretation of RDTs. Click here for file
