# Supplementary material for: Clinical performance of an automated reader in interpreting malaria rapid diagnostic tests in Tanzania
Source: Malar J. 2013 Apr 24;12:141. doi: 10.1186/1475-2875-12-141 (PMC3646688; doi:10.1186/1475-2875-12-141)
Supplement: Additional file 1 — Discordant test Results between Deki Reader and Visual Interpretation of RDTs. [file 1475-2875-12-141-S1.docx]

**Additional file 1. Discordant test Results between DekiReader and Visual Interpretation of RDTs**

| **Sample ID** | **User Interpretation** | **Device Interpretation** | **TBS Microscopy** | **PCR** | **Image of Diagnostic strip** |
| --- | --- | --- | --- | --- | --- |
| 01085 | Neg | Pos | Neg | Neg | 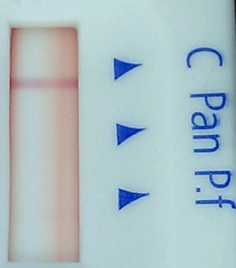 |
| 01260 | Neg | Pos | Neg | Pos | 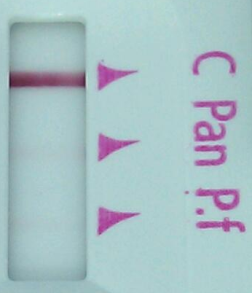 |
| 02154 | Neg | Pos | Pos | Pos | 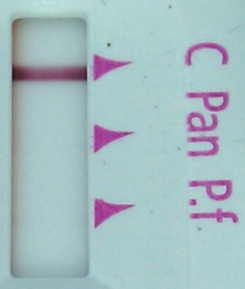 |
| 03222 | Neg | Pos | Neg | Neg | 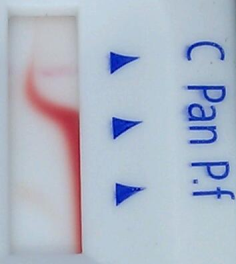 |
| 03351 | Neg | Pos | Neg | Neg | 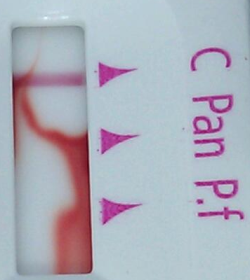 |
| 03399 | Neg | Pos | Neg | Neg | 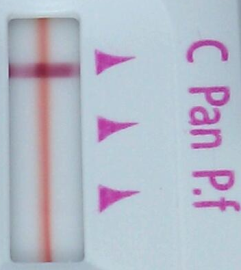 |
| 04010 | Neg | Pos | Neg | Neg | 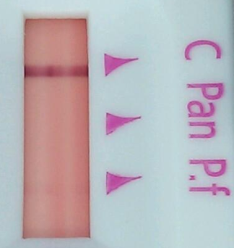 |
| 04189 | Neg | Pos | Neg | Neg | 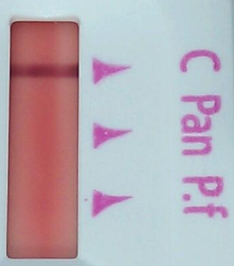 |
| 04218 | Neg | Pos | Neg | Neg | 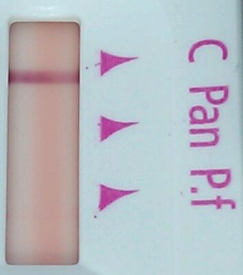 |
| 04250 | Neg | Pos | Neg | Neg | 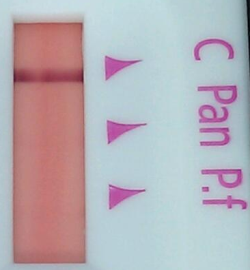 |
| 04311 | Neg | Pos | Neg | Neg | 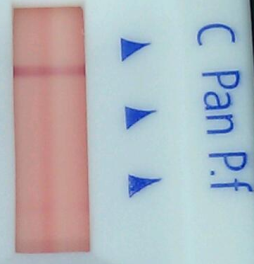 |
